# Dietary supplements to a low protein diet may affect the occurrence of hepatic lipidosis in mink - a strict carnivore

**DOI:** 10.1186/1751-0147-57-S1-O17

**Published:** 2015-09-25

**Authors:** Connie Frank Matthiesen, Anne-Helene Tauson

**Affiliations:** 1 Department of Veterinary Clinical and Animal Sciences, University of Copenhagen, Copenhagen, Denmark; 2 Department of Animal Nutrition and Management, Swedish University of Agricultural Sciences, Uppsala, Sweden

## Introduction

Hepatic lipidosis is a multifactorial disease and may be caused by a number of factors such as low protein provision, feed deprivation, rapid accretion or mobilisation of body fat all resulting in metabolic imbalances.

## Objectives

The objectives were to investigate if supplementation of a low protein diet (LP) with nutrients acting as methyl donors, antioxidants or having insulinogenic properties could lower the incidence of hepatic lipidosis in growing mink from August to November when mortality, caused by hepatic lipidosis, often is high.

## Material and methods

Seventy-two growing mink where allocated into six groups with 6 males and 6 females. The control group was fed a conventional diet (30% of metabolisable energy (ME) from protein) whereas the 5 remaining groups were fed a LP diet (20% of ME from protein) and supplemented with crystalline amino acids (0.8% alanine, 0.5% taurine, 0.5% arginine, 0.5% methionine) or 2.5% dextrose. Balance and respiration experiments were performed and the animals were weighed and blood sampled every third week. The liver and body weights were recorded for all animals.

## Results

Livers from animals with hepatic lipidosis were significantly heavier and contained more fat than livers from healthy animals. The survival rate was significantly higher for the control and methionine groups (100%) than for the dextrose group (75%) and numerically higher than for alanine (92%), taurine (92%) and arginine (83%).

## Conclusion

It can be concluded that our results indicate that the methionine level in a low protein diet plays an important role for the occurrence of hepatic lipidosis.

